# Does Rubella Cause Autism: A 2015 Reappraisal?

**DOI:** 10.3389/fnhum.2016.00025

**Published:** 2016-02-01

**Authors:** Jill Hutton

**Affiliations:** ^1^Department of Obstetrics and Gynecology, The Woman’s Hospital of Texas, Houston, TX, USA

**Keywords:** rubella, congenital rubella syndrome, autism, congenital rubella infection, rubella virus, autistic spectrum

## Abstract

In the 1970s, Stella Chess found a high prevalence of autism in children with congenital rubella syndrome (CRS), 200 times that of the general population at the time. Many researchers quote this fact to add proof to the current theory that maternal infection with immune system activation in pregnancy leads to autism in the offspring. This rubella and autism association is presented with the notion that rubella has been eliminated in today’s world. CRS cases are no longer typically seen; yet, autistic children often share findings of CRS including deafness, congenital heart defects, and to a lesser extent visual changes. Autistic children commonly have hyperactivity and spasticity, as do CRS children. Both autistic and CRS individuals may develop type 1 diabetes as young adults. Neuropathology of CRS infants may reveal cerebral vasculitis with narrowed lumens and cerebral necrosis. Neuroradiological findings of children with CRS show calcifications, periventricular leukomalacia, and dilated perivascular spaces. Neuroradiology of autism has also demonstrated hyperintensities, leukomalacia, and prominent perivascular spaces. PET studies of autistic individuals exhibit decreased perfusion to areas of the brain similarly affected by rubella. In both autism and CRS, certain changes in the brain have implicated the immune system. Several children with autism lack antibodies to rubella, as do children with CRS. These numerous similarities increase the probability of an association between rubella virus and autism. Rubella and autism cross many ethnicities in many countries. Contrary to current belief, rubella has not been eradicated and globally affects up to 5% of pregnant women. Susceptibility continues as vaccines are not given worldwide and are not fully protective. Rubella might still cause autism, even in vaccinated populations.

## Introduction

Rubella “is so mild and its sequelae so severe that any connection between them would hardly be suspected … it put so much distance between itself and its sequelae – at least several months and into the next generation – that it was not detected until epidemiological overconfidence from a long period of quietly getting away with it” (Aycock and Ingalls, 1946). Diligently following the evidence, rubella is likely still “getting away with it.”

After widespread vaccination, the congenital rubella syndrome (CRS) babies almost disappeared from the developed world. Currently less than a handful (usually <2) of cases of CRS occur in the US each year (Dobson, 2014). The US is a widely covered population with regards to rubella vaccination. But perhaps those few straggling cases of CRS each year are just the tip of the iceberg. Maybe rubella does not always cause a full-blown case of CRS easily recognized as it did in the 1960s. Maybe there is a lesser-known outcome: a disorder widely diagnosed today, autism.

Autism has many known causes; yet, even in aggregate these known causes only account for a minority of cases. The majority of autistic cases are idiopathic. Among the known genetic and epigenetic causes of autism, rubella has long been overlooked. Autistic children often share findings of CRS. Neuropathology and neuroradiology findings in autism often hint at a viral etiology. The rubella virus, like autism, crosses many ethnicities in many countries. In some well-developed regions of the world, immigrants from less-developed areas have more children affected by autism, leading to theories of exposure. Contrary to current belief, rubella has not been eradicated and globally affects up to 5% of pregnant women – as detected only by serology, not clinically. Without serological studies of pregnant women, rubella’s presence would not be known. Vaccination is essential, but some women do not seem to respond to the vaccine, and titers can wane over time, leaving older mothers more at risk. Susceptibility continues as vaccines are not given worldwide and are not fully protective. Rubella might still cause autism, even in vaccinated populations.

## Rubella and Autism

In the 1960s–1970s, Stella Chess likened autism in children with CRS to “a chronic infection in which recovery, chronicity, improvement, worsening, and delayed appearance of the autistic spectrum all were found” (Chess, 1977). She studied a group of 243 children with CRS, ages 2.5–3 years old, and using Kanner’s strict criteria diagnosed autism in 10, and partial autism in 8 of them. Diagnosing a total of 18 children affected with autism in the 243 children with CRS, she quoted a prevalence of 7.41%, a prevalence over 200 times that of the general population of the US at that time (Chess, 1971). In a follow-up study of 205 of the original children at the ages of 8–9 years old, including 17 of the initial 18 with autism, she then diagnosed an additional 4 cases. In this follow-up study, her incidence of autism was (17 + 4)/205 or 10.24% (Chess, 1977) using the guidelines of the 1970s to diagnose autism; if she had used the current guidelines, she likely would have diagnosed a greater percentage. While Chess studied the outcome of the 1963–1964 rubella epidemic in New York, two other groups in Houston and Philadelphia likewise followed CRS affected infants.

Desmond and others in Houston reported neurologic symptoms in 81 of 100 CRS cases. At birth, nervous system symptoms included: bulging fontanelle (45%), lethargy (28%), irritability (45%), head retraction, back arching (49%), problems with tone (81%), difficulties swallowing (15%), and convulsions (27%). At 18 months, the surviving group of 64, had neuromotor problems (50%), hyperactivity (25%), stereotyped postures and movements (18.8%), delayed or absent language (75%), autism (12.5%), and hearing loss (45.3%) (Desmond et al., 1969). In reference to theses case series, Stella Chess concluded, “the high prevalence of autism was one consequence of the invasion of the central nervous system by the rubella virus” (Chess, 1977).

In Philadelphia, Lindquist, Plotkin, and peers estimated approximately 1–1.4% of all births had been affected by rubella during the 1960s outbreaks (Lindquist et al., 1965), the largest outbreaks in US history. In 1964–1965 alone, the US saw an estimated 12.5 million cases of rubella, resulting in 2000 cases of encephalitis (or 1 in 6250 cases), 11,250 abortions, 2100 cases of neonatal deaths, and 20,000 cases of CRS. Of the 20,000 cases of CRS: 11,000 (55%) were deaf, 3580 (17.9%) were blind, and 1800 (9%) were mentally retarded (Jacobson et al., 2009). During these largest outbreaks in US history, the association of rubella and autism became apparent, but only through massive numbers of known infected individuals. The strongest argument against a spurious association is the fact the children studied with CRS had prevalence rates of autism over 200 times that of the overall prevalence rate of autism at that time, the 1970s. In 2015, with widespread vaccination in the US, for rubella to remain a causative factor in autism, unknown infected individuals must exist.

## Rubella Teratogenicity

In the 1940s, an Australian ophthalmologist, Norman M. Gregg was the first to suggest rubella as a teratogen of congenital cataracts (Gregg, 1991). His theory was not immediately accepted, but soon became evident as the epidemic spread with the global movement of people during World War II. The 1940s established rubella embryopathy, beginning with the classic triad of cataracts, deafness, and heart defects recall (Dunn, 2007).

Rubella contains a single-stranded RNA (9762 nt), a nucleocapsid, a lipoprotein envelope, and spiky projections E1 and E2. E1 is the major epitope (Lee and Bowden, 2000; Bantavala and Brown, 2004). Humans are the only natural host; the human host receptor is unknown (Webster, 1998; Lee and Bowden, 2000; Nguyen et al., 2013). Once rubella invades a cell, an inclusion body (50–70 nm) develops in the cytoplasm. These inclusions appear to be virus-modified lysosomes, which are often linked to mitochondria and endoplasmic reticula. Rubella utilizes energy from the mitochondria, and incorporates cardiolipin from the inner lining of the mitochondria into rubella virions (Lee and Bowden, 2000). Rubella’s use of cellular organelles likely stunts cellular growth. *In vitro* studies show stagnant cell division. Rubella slowly replicates causing little apoptosis and a steady presence (Lee and Bowden, 2000). Rubella also induces much larger cytoplasmic inclusions in the 5–8 μm range identified as “macrolysosomes” (Kouri et al., 1974). Rubella disrupts the cytoskeleton of cells by depolymerizing actin (Lee and Bowden, 2000), a molecule necessary for mitosis, signaling, and migration; without actin, cells cannot easily divide or position themselves for proper embryonic development.

A mother infected with rubella develops viremia in 5–7 days, and possibly a rash at 16–20 days, though 50–75% of cases are without known exposure and without symptoms, or are subclinical (Webster, 1998; Lee and Bowden, 2000; Dobson, 2014). Congenital rubella infection (CRI) is viremia spread to the fetus, but does not designate whether the fetus has defects of CRS (Dobson, 2014). The highest chance of fetal infection occurs if a mother is infected with rubella at <12 weeks or >36 weeks gestation, with a range of 25–100% throughout pregnancy (Webster, 1998). Fetal infection (CRI) can be chronic and often persists after birth; babies can have prolonged shedding of virus for up to a year after birth, but rarely up to and past 2 years (Dobson, 2014). This persistent shedding aids the rubella contagion. Concerning CRS or defects, Miller et al. studied clinically diagnosed rubella virus exposure at various gestational ages and found that 100% of fetuses infected <11 weeks gestation had defects of CRS; 35% of fetuses 13–16 weeks had defects (deafness), and 0% of fetuses after 16 weeks had defects (Miller et al., 1982). Pregnancies with rubella infections clinically diagnosed in the second and third trimesters had a higher percentage of intrauterine growth restriction, but otherwise the babies did not have defects at birth (Dobson, 2014). The placenta was often small, resulting in placental insufficiency with fetal intrauterine growth restriction in 25% (Webster, 1998; Lee and Bowden, 2000) and birth weights <2500 g in 23% of cases with CRI (Dobson, 2014). Data on CRI and CRS reflect 1960s–1970s studies consisting of rubella infection in pregnancy of never-vaccinated non-immune hosts, with clinically apparent cases. Much of this data have not been updated in the past 40–50 years.

How exactly rubella induces birth defects is not entirely known, but certain factors suggest probable mechanisms. Rubella infects the placenta through its vasculature and then may infect any part of the fetus. In the placenta, pathology of aborted early gestations shows necrotic foci, and later gestations shows more vasculitic and inflammatory changes (Webster, 1998). Subsequent to vasculitis, rubella spreads to other tissues both directly though disrupted vascular lumens, and indirectly through emboli of infected cells and circulating infected mononuclear cells (Webster, 1998; Nguyen et al., 2013). Rubella does not infect all fetal cells, but sporadically infects about 1 in 1000 to 1 in 100,000 cells; and then continues to exist within that cell’s progeny (Webster, 1998; Lee and Bowden, 2000). In this manner, rubella may evade the immune system. Using immunofluorescence, pathology has shown infected organs have scattered foci of infected cells, compact and with discrete borders (Webster, 1998; Lee and Bowden, 2000). *In vitro*, human astrocytes are “selectively and heavily infected” (Webster, 1998). Because not all cells are infected, most cells and structures are grossly normal with only foci of abnormal cells and their clones. Hence, rubella leads to foci of stunted and displaced tissues (Lee and Bowden, 2000). Another possible cause of teratogenicity is cell destruction caused by inflammatory changes and or autoimmune processes (Webster, 1998). These processes occur later and are more notable in rubella infections of gestations >20 weeks and after birth (Lee and Bowden, 2000).

## CRS and Rubella Sequelae

Because the virus can infect any part of the developing fetus, any organ can be affected. The more common anomalies attributed to rubella or the “A” findings include: hearing loss, heart defects, cataracts, and pigmented retinopathy. The lesser attributed signs or “B” findings include: microcephaly, developmental delay, hepatosplenomegaly, purpura, jaundice, meningoencephalitis, and radiolucent bone disease. CRS is diagnosed with an A finding and lab confirmation in the newborn with any of the following: serologic rubella-specific IgM, persistent rubella-specific IgG, and polymerase chain reaction (PCR) positive for rubella or isolated rubella virus. CRI is diagnosed by lab confirmation only. CRI signifies infection *in utero*, but does not imply any syndrome or defects. Probable cases of CRS have 2A findings, or 1A and 1B, but no lab confirmation. Suspected cases of CRS have B findings only (Dobson, 2014; Pitts et al., 2014).

As children develop, behavioral findings associated with CRS may emerge and include: hyperactivity, spastic diplegia, absent or delayed language, or autism (Desmond et al., 1969; Webster, 1998). Late changes include diabetes mellitus type 1 (DM1), thyroid disorders, and hypertension (Dobson, 2014). After the Australian epidemic of rubella in the 1940s, over 40 CRS affected individuals were followed >50 years. Concerning DM1, 20% developed DM1 by age 25, and 22% by 50–60 years. More recent data put the estimate of DM1 in CRS individuals at 1% by adolescence. Thyroid disorders occurred in 5% of survivors (Dobson, 2014). Theories regarding the late findings include autoantibodies confusing rubella and self isotopes (Dobson, 2014), and rubella-specific immune complexes circulating and causing proteinuria, abnormal glucose tests, thyroiditis, and seizures (Coyle et al., 1982). Another late, but rare finding in CRS is progressive rubella panencephalitis (PRP), a slow and often fatal inflammatory brain disorder (Dobson, 2014). Early defects, behavioral changes and later complications were all described several decades ago, the result of perinatal rubella infections in mothers without any prior contact with rubella.

## CRS Findings Occur with Autism

Congenital rubella syndrome comorbidities: the early effects of Gregg’s embryopathy, then behavioral changes and finally late consequences are seen in autism. Looking at Gregg’s embryopathy or the three most attributed findings with CRS: deafness occurs in 60% of CRS cases, heart defects occur in 45% of CRS cases, eye findings of cataracts occur in 25% of CRS cases, and pigmented retinopathy occurs in 5% of CRS cases (Dobson, 2014). In trying to link all of these findings together with autism, there are no current studies past the original studies reported from New York, Houston, and Philadelphia. Looking at the three areas of hearing, heart and vision defects each individually with autism a few good studies emerge. Hearing deficits and auditory hypersensitivity are common in children with autism. A review article quotes a study by Rosenthal et al. diagnosing moderate to severe hearing loss in 7.9%, and severe hearing loss in 3.5% of a group of 199 children with autism; a study by Gillenberg et al. diagnosing hearing loss in 13.3% of a group with autism; a study by Klin estimating hearing abnormalities, both of central and peripheral origin, in 33–46% of persons with autism; and a study by Jure demonstrating autism in 5.3% of hearing impaired individuals (Hitglou et al., 2010). Regarding congenital heart disease (CHD), a study of children, 420 with CHD and 108,048 without CHD, showed that the group with CHD had more diagnoses of autism; OR 4.6 (95% CI 1.9–11) (Razzag et al., 2015). Lastly, visual changes noted with autism do not include cataracts, but include more refractive errors, strabismus, and perceptual differences. Certain studies show an increase in strabismus in individuals with autism, ranging from 20 to 50% (Simmons et al., 2009). A more recent study investigated ophthalmological exams of a group of 324 patients with autistic spectrum disorder (ASD) uncovering 22% with refractive errors and 8.6% with strabismus (Kabatas et al., 2015). The common features of CRS, including hearing loss, heart defects, and visual changes, are also seen in autism.

As far as behavioral changes noted with CRS: hyperactivity, neuromotor problems, or spastic diplegia, absent or delayed language, and autism (Webster, 1998; Dobson, 2014); autism is built-in as one of the findings, and delayed speech is a factor in the diagnosis of autism. Only hyperactivity, and neuromotor problems or spastic diplegia will be investigated for current associations with autism. Severe hyperactivity and the diagnosis of attention deficit hyperactivity disorder (ADHD) are frequent in subjects with autism, applied to 33% of individuals with ASD (Carlsson et al., 2013). Spastic diplegia is commonly categorized with cerebral palsy (CP). A 2008 study looking at a cross-section of 8 years old noted 6.9% of 8 years old with CP had autism; a bit paradoxically 18.4% of 8 years old in the non-spastic CP group had autism (Christensen et al., 2014). A 1997 study noted CP in 2.9% of children with autism (Fombonne et al., 1997). Behavioral changes noted with CRS definitely overlap with autism, and the overlay of autism with CP hints at a cerebral insult in the prenatal period.

Late manifestations of CRS mainly include endocrinopathies such as DM1 and thyroid disorders, but also hypertension and vascular disease. Is there any evidence of these diagnoses in the autistic population? A 2012 study out of the Boston area examined comorbidities in 14,000 hospital patients with autism. They stratified their data by ages 0–17 years and 18–34 years to see which diagnoses increased with age and found that DM1 increased from 0.67 to 2.08% (Kohane et al., 2012). Investigating endocrinopathies, DM1 increases over time in a small percentage of patients with autism. Looking at vascular disease in autism, which has been ascribed to CRS, a study of 108 ASD patients followed into adulthood found 34.9% to be obese, 31.5% to have hyperlipidemia, and 19.4% to have hypertension. The only one of these findings significantly above the control cohort was hyperlipidemia with an OR of 2.0 (Tyler et al., 2011). Hypertension, though seemingly common in persons with autism, is not particularly increased above the general population. Late diagnoses seen in a small percentage of individuals with CRS are merely hinted at in autism, as though autism might be a diminutive form CRS.

Lastly does PRP, a declining neurological disorder with symptoms typically of dementia, ataxia and/or seizures (Kuroda and Matsui, 1997), have any correlation to autism? For the true diagnosis of PRP, the answer is clearly no. However, autism has a regressive or sometimes called disintegrative form in which a normally developing child will regress, often losing language and motor skills, and/or having seizures. In autism, epilepsy ranges from 19 to 33% with some subclinical cases only picked up by EEG, and with a higher percentage of epilepsy existing in the disintegrative/regressive form of autism (Muhle et al., 2004; Kohane et al., 2012). Regressive autism shares symptoms with PRP, but unlike PRP is not often described as fatal. Once again autism, the disintegrative form for this comparison, is perhaps just a wisp of the full outcome of CRS, in this case PRP.

## Neuropathology in Rubella and Autism

In these next paragraphs, first CRS will be examined for neuropathology findings and then autism will be similarly examined for neuropathology findings. A few points: neuropathology from CRS cases is often from aborted fetuses after a prenatal diagnosis. As there is no prenatal diagnosis for autism, there are no such studies available for comparison. Furthermore, neuropathology cases of CRS mostly come from severe, fatal cases in infancy. Neuropathology cases of autism are less prevalent; and as autism is not often fatal, the specimens are not from severe cases, but rather from incidental deaths in adulthood at ages extending beyond 60 years old.

Neuropathology of CRS is often studied on specimens electively aborted after a prenatal diagnosis. CRS findings are consistent with viral invasion and replication within the brain, but of few cells overall, not all cells (Webster, 1998). Cerebral vascular lesions and hemorrhages occur in early embryos infected with rubella (Webster, 1998). Of 67 aborted fetuses with CRS, no gross abnormalities were seen, but 68% had microscopic changes and 32% had no microscopic changes. Some chorionic cells had eosinophilic cytoplasmic inclusion bodies (Tondury and Smith, 1966). In 2015, a study from Vietnam of three aborted fetuses with CRS, showed no encephalitis, but rubella RNA was detected by PCR in the fetal CNS (Nguyen et al., 2013). As CRS is estimated to infect 1 in 1000 to 1 in 100,000 cells of the fetus, in some pathology specimens minimal changes are seen (Webster, 1998).

Neuropathological studies of children severely affected with fatal cases of CRS often show extensive changes. Rourke studied the brains of nine infants <1 year of age and noted leptomeninges with fulminant degeneration (Rorke and Spiro, 1967). Pathology of youths approximately ages 8–20 who died after PRP demonstrated significant volume loss with white matter destruction, vasculitis, cerebellar atrophy, fulminant necrosis, and old infarcts. Theories on the cause often include inflammatory insults, cerebral vasculitis, and to a lesser extent direct viral inhibition of growth and destruction of cells (Kuroda and Matsui, 1997; Webster, 1998; Sawlani et al., 2013).

The other major finding in CRS neuropathology is that of cerebral necrosis, often in the periventricular areas. Rourke reported necrotic foci in deep white matter and gray nuclei (Rorke and Spiro, 1967). Rubella often follows vascular routes causing ischemia along their distributions, especially the small penetrating vessels (Webster, 1998). Emboli of necrotic cells in brain arterioles and desquamation of vessel lumens were visualized (Tondury and Smith, 1966). Cerebral blood vessels show focal areas of destruction with thickened walls and narrowed lumens (Webster, 1998). Pathology suggests the virus causes vasculitis and cellular emboli. Although many infants died of cyanotic cardiac lesions which might also result in emboli, the vasculitis and cerebral emboli are attributed to the rubella virus because similar vascular lesions are seen in some embryonic brains from first trimester abortions (Webster, 1998). In summary, the neuropathology of CRI/CRS cases may reveal no abnormalities as seen in some embryonic cases; or may show degenerative changes, cerebellar atrophy, infarction (especially periventricular), and cerebral vasculitis with narrowed lumens.

Looking at autism, neuropathology studies are infrequent and incidental. Though brains from CRS cases and from autism share the similarity of many normal appearing brains, there are many dissimilarities. Instead of degeneration and neuronal loss as seen in CRS, studies of autism suggest increased brain growth, especially in the forebrain, in the first 1–2 years of life with 20–25% of children with autism having macrocephaly (Wegiel et al., 2010; McFadden and Minshew, 2013). In the frontal lobe, neurons appear smaller and more densely packed, with abnormal organization of the minicolumn, a vertical unit of cortex organization (Acosta and Pearl, 2003; Wegiel et al., 2010; Williams and Casanova, 2011; McFadden and Minshew, 2013). Poor differentiation is often seen at the white-gray matter junction (especially in the periventricular area) thought to be due to excess remnant cells. Autistic brains’ growth has been studied from infancy well into adulthood. In CRS, most brains are studied only from neonates to infants. In autism, abnormal neuronal connections have been implicated and mapped with newer high definition fiber tracking technology aided by improved functional imaging. Though widely speculated, increased shorter connections and decreased longer connections in autistic brains have been both proven and unproven (Sundaram et al., 2008; Williams and Casanova, 2011; McFadden and Minshew, 2013). Decreased pruning of synapses is evidenced and accepted by most (Williams and Casanova, 2011; McFadden and Minshew, 2013). Some brains show temporal horn dilation, and cells in the temporal area have been shown to have an abundance of dendritic spine densities (McFadden and Minshew, 2013). Many studies show decreased Purkinje cells in the cerebellum, thought to generate, migrate and die before birth with inflammatory microglial cell activation (Tondury and Smith, 1966; Bailey et al., 1998; Acosta and Pearl, 2003; Whitney et al., 2009; Williams and Casanova, 2011; McFadden and Minshew, 2013). The prenatal loss of Purkinje cells in the cerebella of those with autism unlikely correlates with cerebellar atrophy seen in those with PRP, as cerebellar atrophy would seemingly result from an overwhelming inflammatory attack of the brain well after birth. Both suggest inflammation, but in general, studies of brains from autistic subjects do not show infarction or vasculitis. CRS demonstrates more ischemic and degenerative changes, and autism elucidates more changes in growth and connectivity.

Autistic brains also have their own findings of ectopic brain tissue and one case with cellular inclusion bodies. In a case series of six brains, of a 4-year old five individuals in their 20s, with more severe cases of autism (Bailey et al., 1998), some interesting findings emerge with possible links to CRS or perhaps other heterogeneous causes of autism. The brains weighed more than average brains of like aged children, but the brain stems and cerebella weighed less. Findings of one or more children showed hyperconvoluted temporal lobes, blurred white-gray matter areas, ectopic gray matter, ectopic neurons in cerebella and lateral to the olives, inferior olive malformations, and decreased Purkinje cells in cerebella. One child had cytoplasmic eosinophilic inclusions in cerebellar Purkinje cells of about 7 μm and staining deeply with Luxol fast blue and cresyl violet. Cytoplasmic inclusions frequently signify a viral infection, but the authors only pondered the inclusions hinted at the varied causes of autism (Bailey et al., 1998). Another case series of 13 brains from autistic individuals of ages ranging from 4 to 60 years showed subcortical-periventricular, hippocampal and cerebellar heterotopias in 4 of 13; cerebellar dysplasia in 4 of 13; cerebellar malformation in 5 of 13; and floculonodular cerebellar dysplasia in 6 of 13. They theorized problems in maturation and migration and pointed to genes related to migration and cytoarchitecture (Wegiel et al., 2010). Thinking back to rubella’s infection of sporadic cells and their clones, rubella’s scattered foci of infected cells, and rubella’s ability to depolymerize actin – a molecule with functions in cell shape, motility, signaling, mitosis, junctions and migration of cells in embryogenesis (Webster, 1998; Lee and Bowden, 2000) – could explain findings in the autistic brain of heterotopic foci of immature neurons, aberrant connections, and the loss of Purkinje and temporal lobe cells (Figure S1). Not to overlook the many dissimilarities in neuropathology studies of CRS and autism, but one wonders what adult brain specimens of mildly affected CRI/CRS might show neuropathologicaly with newer fiber tracking technology.

## Neuroimaging in Rubella and Autism

Neuroimaging of CRS individuals includes adolescents and adults, and surprisingly their scans have similarities to neuroimaging of autistic individuals; neuroradiology studies of CRS are less common as more prevalent CRS cases existed before magnetic resonance imaging (MRI) or positron emission tomography (PET) came into existence, and also neuroradiology studies of autistic children probably have a bias toward higher functioning individuals who can tolerate such imaging, or rather, being in the machines with loud noises while able to follow commands. MRI was not available during the larger epidemics of rubella in the developed world, but there are a few case series and case studies of brain MRI results in CRS survivors. A case series by Lane et al. imaged brains of 11 CRS affected individuals and showed white matter hyperintensities in 6 of the 11 studied. White matter lesions, associated foci of ischemic necrosis, and small associated cysts showed involvement of penetrating vessels in the deep white matter and represented ischemic brain damage along the path of vessels affected by the rubella virus. Periventricular areas and basal ganglia were frequently involved (Lane et al., 1996). There are several case reports of neurological MRI findings in CRS children. An MRI of a case documented in 2013 showed cystic encephalomalacia, periventricular calcifications, atrophic changes in the temporal lobe, and lesions consistent with gliosis and demyelination (Sawlani et al., 2013). Another case from 2013 showed “diffuse leukoencephalopathy with subcortical anterior temporal cysts, which showed spontaneous improvement during a period of 3 years” (Severino et al., 2014). A suspected case of CRS documented in 2014, in a boy also diagnosed with autism, showed cystic encephalomalacia in “the subcortical white matter of the left posterior temporo-parietal occipital lobes and left basal ganglia” (Hutton and Hutton, 2014). A computed tomographic (CT) scan of a case published in 2008 showed calcifications in the left corona radiata (Pitts et al., 2014). MRI and CT of a CRS case from 2003 showed calcifications in the periventricular area and basal ganglia (Numazaki and Fujikawa, 2003). A CT scan of a case of CRS in 2000 showed microcephaly, periventricular leukomalacia, calcifications, and subcortical atrophy (Bullens et al., 2000). To summarize, brain MRI findings of CRS affected individuals often show white matter hyperintensities, calcifications, leukomalacia, and atrophic changes, in areas described along the distribution of deep penetrating vessels at the white-gray matter junction, periventricular areas, temporal areas, and basal ganglia.

Magnetic resonance imaging and PET scan studies of individuals with autism are numerous and largely have a bias toward higher functioning individuals. Repeating a case listed above, and serving as a reminder of findings, a suspected case of CRS in a boy also diagnosed with autism showed encephalomalacia especially in the temporal area and basal ganglia, and also prominent perivascular spaces (Hutton and Hutton, 2014). A study from France of 69 ASD children showed 52% (36 of 69) had normal MRIs, and 48% (33 of 69) showed findings of: abnormal white matter signals (19 of 69), dilated Virchow–Robin/perivascular spaces (12 of 96), and temporal lobe abnormalities (20 of 69). The authors even state that the white matter hyperintensities found in the posterior horns of the lateral ventricles were similar to those of “periventricular leukomalacia, metabolic disorders, viral infections or vascular disorders”; white matter abnormalities have been seen in CP; and that some deep white matter lesions (area of deep penetrating vessels) cause suspicion for “a vascular etiology.” They further theorized that the temporal lobe changes could explain epilepsy seen in many individuals with ASD. They concluded that findings were non-specific overall, but white matter hyperintensities and temporal lobe abnormalities were seen in 26% of their cases (Boddaert et al., 2009). Of all the studies, this one reports MRI findings very similar to those seen with CRS, most notably the hyperintensities seen in white matter suggestive of viral or ischemic changes, and temporal horn abnormalities. Another French study of 77 children with autism, showed loss of gray matter in the superior temporal sulcus in 21 cases, and PET scans showed decreased blood flow in the same region (Brunelle et al., 2009; Zilbovicius et al., 2013). Many PET scans of autistic brains show decreased flow in cerebral vessels, a finding akin to rubella affected brains. PET scans on 15 children with autism and infantile spasms showed 13 had decreased metabolic activity in the temporal lobe, 9 in the frontal lobe, and 7 in the parietal lobe (Dilber et al., 2013). A PET study on autistic subjects showed decreased perfusion in the temporal cortex, insula, mediofrontal cortex, left inferior frontal cortex, and left middle frontal cortex. No areas had increased perfusion (Ohnishi et al., 2000). In another PET study from Japan, 22 individuals with ASD had decreased blood flow to bilateral latero-temporal and dorso-medio-lateral frontal areas; the left side being worse than the right (Hashimoto et al., 2000). Diffusion tensor imaging studies also reveal changes in the frontal, temporal, and parietal lobes (Sundaram et al., 2008; Ke et al., 2009). In theory, areas with decreased perfusion are linked to increased hypoxia and lower intelligence (Sharma et al., 2013; Zilbovicius et al., 2013). Referring back to MRIs of individuals affected by CRS, many show periventricular leukomalacia and calcifications suggestive of end-artery occlusion, and atrophic changes in the temporal area of the brain. There are no PET studies of children with CRS, yet areas in the brain with less perfusion and metabolic activity seen in autistic persons correlate to areas affected by CRS. Pathology of CRS cases typically demonstrates that rubella causes vasculitis, narrowed lumens and brain ischemia at the deep penetrating arteries of the brain (Webster, 1998). Possibly, then the PET studies of autistic individuals demonstrating decreased perfusion with brain hypoxia could be a result of these vessel changes and or ischemic changes caused by rubella virus (Figure S1).

## Neuroimmunology in CRI and Autistic Individuals

Growing evidence in the field of neuroimmunology points to an immune mechanism in the cause of autism, either innate with chronic activation, a maternal response which attacks the fetal brain, or perhaps an autoimmune reaction. Lab techniques and serology tests have progressed in the last 40 years, so that immune studies are more advanced and encompassing with regards to autism. Embryos with CRI, and CRS infants, pathologically show more cerebral vasculitis than encephalitis. However, neurological aspects also implicate immune system involvement especially in later gestations (past 20 weeks) (Lee and Bowden, 2000), in CRI cases with encephalitis sometimes lasting for years, and in PRP (Webster, 1998). Though early embryos rarely show evidence of inflammation, those after 20 weeks often show increased IgM, IgG, IgA, natural killer cells, T cells, and interferon (Lee and Bowden, 2000). A case of a boy who died from PRP showed increased IgA and IgG specific to rubella in the brain as opposed to sera. IgA was increased 1- to 60-fold, particularly in the parietal lobe and cerebellum; and IgG was increased 8- to 27-fold, particularly in the parietal and temporal lobes. The authors postulated the immunoglobulins were produced in the brain to bind up antigen, and hence these were the areas most affected by the virus (Wolinsky et al., 1982). CRS is often described as a persistent infection in infancy (Dobson, 2014; Bantavala and Brown, 2004). Autism is described as a persistent immune reaction, most notably in the frontal and temporal lobes (Michel et al., 2012). Evidence of inflammation in brains includes the chronic activation of microglial cells is seen in both autism (Goldani et al., 2014) and CRS (Lindquist et al., 1965). In considering immune modulators, many tests for cytokines were not available 30–50 years ago when more children were diagnosed and studied with CRS; information as such is minimal, though alpha-interferon (INF-alpha) was shown to be increased in fetuses with CRI (Lee and Bowden, 2000). With autism, multiple studies show increased inflammatory cytokines in the sera of those with autism. Such inflammatory cytokines include: interleukin-1beta, interleukin-6, interleukin-8, tumor necrosis factor alpha (TNF-alpha), and gamma-interferon (INF-gamma); and with many of these cytokines, the higher their concentrations, the more severe are the symptoms of autism (Ashwood et al., 2011; Michel et al., 2012; Xu et al., 2015). On the other hand, the anti-inflammatory cytokine transforming growth factor-beta is decreased in persons with autism (Michel et al., 2012). TNF-alpha is postulated to have an effect on pruning of synapses within the brain (Michel et al., 2012). Recent data on cytokines and rubella infection are lacking, especially *in vivo* studies. An *in vitro* study “interrogated” 33,000 genes before and after infecting human cells of fetal and adult lines with rubella, demonstrating upregulation and downregulation in 632 and 516 genes, respectively of multiple modalities: inflammation; cell growth, division, structure, adhesion, and signaling; and unknown functions (Adamo et al., 2008). Again in CRS, given the era of in depth investigations, INF-alpha is the only documented immune modulator known to be increased. Investigating inflammatory markers in CRI needs much advancement, with the aforementioned article being a good start.

Maternal infection has been implicated in autism. In a study of possible vertically transmitted viruses in the brains of subjects with autism, the 15 autistic brain specimens had more PCR evidence of BK virus, JC virus and simian virus 40 combined than the 13 control brain specimens (Lintas et al., 2010). They did not include rubella virus in their search. Women with anti-influenza antibodies in the first half of their gestation have a three to seven times increase of having a child with autism (Shi et al., 2009). Pregnant mice given influenza mid-gestation had offspring with decreased Purkinje cells in the cerebella, heterotopic Purkinje cells, and delayed migration of neurons. The same outcome happened if the pregnant mice were given dsRNA poly(1:C), an immune stimulant (Shi et al., 2009). More recently, similar findings are emerging in a rhesus monkey model (Weir et al., 2015). Unfortunately, there is no current animal model for CRI. In 1967, a study demonstrated teratogenicity of rubella in macaques and baboons (Wilson and Gavan, 1967). An animal model might help elucidate further pathophysiology, but animal models have not been developed. CRS brain lesions may result from maternal inflammation (Shi et al., 2009), and CRS is associated with autism, and was before the diagnosis of autism became expansive and common. In groups of children with CRS, 7.41–12% also had autism (Desmond et al., 1969; Chess, 1971, 1977); the 7.41% estimate was 200 times the prevalence of autism in the general population in the 1970s.

## Genetics of Autism and Rubella Immunity

For years, scientists studying autism have searched for an underlying genetic pathway. Genetics was strongly implied by a twin study showing 60% of monozygotic (MZ) twins were concordant for autism while 0% of dizygotic (DZ) twins were concordant for autism. When expanded to look at the broader spectrum of autism, 92% of MZ twins and 10% of DZ twins were concordant (Bailey et al., 1995; Muhle et al., 2004). Genetic studies began screening families with members affected by autism, families with *de novo* cases of autism, and a slew of genes with any possible relation to autism. These paths have been quite divergent, investigating over 500 different genes, with several genetic loci on every chromosome (Goldani et al., 2014). More recent studies estimate environmental factors likely account for at least 58% of autism (Michel et al., 2012; Goldani et al., 2014). Many MZ twins share placental blood circulation, such that an infection would be more likely to affect both MZ twins than DZ twins (Michel et al., 2012), though there is a case study of CRI in MZ twins where one twin escaped rubella infection (Wang et al., 1990). Furthermore, genetics do not account for the first-born preponderance, or male predominance in autism with the male to female ratio in autism being about 4:1. The Boston study cited above, also found the typical male to female ratio of 4:1, and looking at genetic disorders in their autism population they found 0.5% had Fragile X, 0.8% had Tuberous Sclerosis (TS), and 0.9% had Down Syndrome (DS) (Kohane et al., 2012). The genetic disorders of fragile X, TS, and DS closely mimic an older study by Fombonne in 1997, showing of 174 children with autism 2.9% had chromosomal abnormalities including Fragile X, 1.1% had TS, 1.7% had DS, and 0.6% had neurofibromatosis (Fombonne et al., 1997). Boys with fragile-X and autism led to many gene investigations on the X chromosome. Though autism affects a preponderance of males, autism has not been found to be X-linked as male to male inheritance occurs (Muhle et al., 2004). Possibly unrelated, but notable, all 20 cases of PRP described from 1974 to 1997 have been males (Kuroda and Matsui, 1997). No one is certain yet why autism affects so many more males than females. Genetic studies have not given a good explanation, and most known syndromes causing autism such as TS and DS only account for a small percentage of the autistic spectrum overall.

If 58% of autism is environmental, then what explains the estimated 42% which may be genetic. Perhaps the genetic factor has to do with a susceptibility to rubella. The host receptor for rubella has yet to be determined, which might account for some variation (Lee and Bowden, 2000). Further, individuals can have different responses to the rubella vaccine. A study of twins showed that there is considerable heritability to rubella vaccine response. The ratio of genetic variance to total variance was 45.7% (p. 003) (Jacobson et al., 2009). This and similar studies linked HLA haplotypes and single-nucleotide polymorphisms to changes seen in individual responses to the rubella vaccine with regards to immunoglobulin levels, immunoglobulin avidity, and production of certain cytokines (Jacobson et al., 2009; Kennedy et al., 2014; OvsyannikovaI et al., 2014a,b). Vaccinated women reinfected in pregnancy may have a defect of immunity such that they do not make effective antibodies. Though they show some evidence of immunity, they are actually susceptible (Best et al., 1989). Consequently, the rubella virus, still circulating the globe, with no country completely eradicating the virus, infects non-immune persons, including those incapable of mounting strong immune responses.

## Lack of Rubella Antibodies in Individuals with CRS and Autism

Peculiar to CRS survivors is often their lack of immunity to rubella. *In utero*, the fetus has an immature immune system, and if CRI occurs early, then the fetus will not recognize the virus as foreign. CRI individuals develop a tolerance to rubella, in particular the E1 epitope (Dobson, 2014; Mauracher et al., 1993; Hyde et al., 2014). With CRI, IgG to rubella is increased at birth and mostly reflects maternal antibody. If months later, IgG to rubella continues to be elevated, then the infant has developed an immune response to the rubella pathogen; and persistently elevated IgG to rubella (before any given vaccination) is one possible lab diagnosis of CRI. Interestingly, in 10–20% of CRI infants, the rubella-specific IgG will drop to undetectable levels, and cannot be stimulated by boosters of rubella vaccine (Dobson, 2014). This immune tolerance may contribute to viral persistence (Webster, 1998; Bantavala and Brown, 2004). Two studies of antibodies in children with autism yielded compelling results. Of 15 children with ASD, 5 of 13 had undetectable antibodies to rubella despite vaccination; in comparison, 8 neurologically typical (NT) children all had antibodies (Stubbs, 1976). Another study which grouped 33 classic autism children and 26 regressive autism children, found 15 of these 59 combined children had low or no antibodies to rubella; as opposed to 2 of 49 in the control group, a combination of 24 children with Tourettes syndrome and 25 NT children (Libbey et al., 2007). Yet, in another study of 31 children with ASD and 29 NT children, the antibodies to rubella were similar in both groups (Gentile et al., 2013). Combining these studies, 20 of 103 children with autism lack antibodies to rubella, similar in ratio to those with CRS lacking antibodies to rubella.

## Rubella Vaccine Effectiveness

With rubella vaccination, a reported 92–98% of individuals will respond with adequate antibodies; however, the vaccine is a live attenuated virus and must be kept cold (the cold chain). With the more usual handling, the response is 86–97% (Jacobson et al., 2009). Lack of immune response is considered primary vaccine failure, and as above ranges from 2 to 8%, or 3 to 14% (with less than perfect handling of the vaccine). Secondary failure is when an individual becomes infected after having prior immunity. In many countries, a second vaccine is given to help overcome primary and secondary failures. Concerning rubella vaccination, secondary failure is also documented. In Italy, 9.8% of females were reinfected within 5 years of vaccination with wild-type rubella (Banerji et al., 2005; Jacobson et al., 2009). In a 1990 study from California, 43% of congenital rubella occurred in moms who had prior rubella vaccinations (Jacobson et al., 2009), about that time the US vaccine schedule added a second MMR. Secondary failure or reinfection with rubella after vaccination is thought to be a rare event, and even rarer to cause a child with CRS, yet there are several documented cases. A mother vaccinated 4 years before becoming pregnant, with a rubella titer of 1:64, had a child with CRS (Miron and On, 1992). A mother vaccinated 7 years prior to the birth of her child had a child with CRS (Enders et al., 1984). A mother with repeated evidence of immunity had a child with CRS; the child had low immunoglobulin levels (Weber et al., 1993). In another case, the mother had a rubella IgG titer of 425 IU/ml at 12 weeks. Initially, this titer was classified as immunity; however, after the birth of her child with CRS, the titer was clarified as an immune response to rubella infection (Banerji et al., 2005). A study evaluating reinfection of mothers giving birth to babies with CRS noted that 8 of 16 of the mothers had rubella titers >15 IU/ml, and even >25 IU/ml (Bullens et al., 2000). These authors could not suggest a lower titer to fully protect against rubella reinfection. Since that study in 2000, several more cases of CRI/CRS have been reported, as born to: a woman with an anti-rubella titer of > 1:16 (Aboudy et al., 2000); a physician mom with a low-titer early in pregnancy and likely infected at work in a hospital with a large immigrant population (Hutton and Hutton, 2014); a woman immunized 14 years prior with a pre-pregnancy titer of 1:16 and a 9-week titer of 1:512 (Ushida et al., 2003); a woman with an early anti-rubella titer interpreted as “immune” early in pregnancy (Numazaki and Fujikawa, 2003); a woman vaccinated 5 years before the birth of her child with a documented anti-rubella titer >400 IU/ml 5 years before her pregnancy to which the authors state she must have had a quickly waning titer or low avidity antibodies (Pitts et al., 2014); and a mother with an early pregnancy anti-rubella titer >15 IU/ml and HIA 1:1024 (Sawlani et al., 2013). To summarize these cases of reinfection in pregnancy, several occurred after vaccination within 4–7 years, with anti-rubella titers >15 IU/ml, and with high early pregnancy titers initially thought to reflect immunity, but in hindsight reflecting reinfection.

At the first prenatal visit, a rubella titer is drawn, even in those having completed vaccination schedules to rubella, so that women whose titers have waned can receive booster rubella vaccinations (Measles, Mumps Rubella vaccine – MMR) after delivery; thus concluding that some women with documented prior vaccination are expected to have low titers. How fast do titers drop? Several studies have tried to answer this question. In Taiwan, rubella vaccination was implemented in 1986. A study checking prenatal titers from 2002 to 2008 revealed 6.5% of women had negative titers (<10 IU/ml) and 8.7% had low titers (10–20 IU/ml), totaling 15.2% with negative or low titers, and with a mean decay rate of −0.77 IU/ml per year (Lin et al., 2012). In Finland, children vaccinated at 14–18 months and then at 6 years were followed, and titers were measured in those vaccinated up to 20 years after initial vaccination. Though all had detectable antibodies at 20 years, 36% had titers <15 IU/ml and 17% had titers <10 IU/ml. Of Finnish mothers in their early 20s, about a third have low levels of antibody to rubella.

Some studies show the possible influx of natural immunity. In Finland, of 178 children aged 14–18 months, 1.1% were immune to rubella before receiving any rubella vaccine. In Central Europe, 224 vaccinated adolescents were followed. After the first vaccine, 92.1% were immune. Titers measured before the second vaccine was given showed at three times increase in 7.8% of those with low titers <40 IU/ml, suggesting a “secondary immune response.” After the second vaccine, 100% were considered immune. An annual decay rate of −2.9% was calculated (Kremer et al., 2006). In Sweden, the 1973 vaccine schedule included one dose of rubella vaccine, and the 1982 schedule included two doses. They followed 486 girls up to 16 years after vaccination. At 16 years, 22% had titers below 1:16 and 6% lacked antibodies, totaling 28%. Mean titers in the 1st, 8th, and 16th year after vaccination were 1:110, 1:34, and 1:18, respectively. At 8 years after vaccination, they found a fourfold rise in 36% of participants, and only in 1% of participants in the following 8–16 years, suggesting natural immunity occurs more frequently at younger ages (Christenson and Bottiger, 1994). Historically, this may not have changed much in the US before widespread vaccination, rubella infections were most commonly seen in 5–9 years old (Lee and Bowden, 2000). In the Netherlands, after following rubella immunity for 32 years, they concluded that titers decrease more quickly after one vaccine than two vaccines, and higher antibody titers are maintained in individuals following natural infections (Smits et al., 2014). Natural infection with rubella produces higher antibody levels, by 5–10 times, than vaccination (Kremerrr and Muller, 2005). There is no worldwide consensus on what titer constitutes rubella non-immunity. Those countries using enzyme-linked immune assays (ELISA) use cut-offs of <10 or <15 IU/ml or sometimes <20 IU/ml; those countries using hemagglutin inhibition assays (HIA) use cut-offs of <1:8 or <1:16. How do ELISA and HIA relate? One article states 1:8 HIA correlates to 15 IU/ml (Robinson et al., 2006); and in another 1:16 HIA correlates to 15 IU/ml (Bullens et al., 2000).

In addition to reinfection being documented by incidental titers or vaccinated mothers giving birth to children with CRS, reinfection has also been documented by a study directly reinfecting those with prior immunity and by a study during an epidemic. Of 29 volunteers with prior vaccination to rubella, reinfected nasally with rubella virus, 6 of 29 (20.69%) were reinfected as diagnosed by a four times increase in titer. No level of anti-rubella titer was found to be protective. A comparison group also with prior vaccination to rubella was challenged intranasally with vaccine-attenuated virus, and none of those exposed showed evidence of reinfection or increased titers (Harcourt et al., 1980). A military study followed 190 men exposed to rubella virus during an epidemic. Of 26 men never exposed or vaccinated, all became infected. Of 15 men with prior vaccination, 12 (80%) showed serology consistent with reinfection. And of 149 men with prior natural immunity, only 5 (3.4%) were reinfected. Not one reinfected individual showed symptoms; of those susceptible, only one in three of those infected showed symptoms, demonstrating the majority of cases were unnoticeable (Horstmann et al., 1970). Subclinical infections are more common in individuals with prior vaccine-induced immunity as opposed to those naïve of prior immunity, and those with natural immunity resist reinfection better than those with vaccine-induced immunity.

## Global Variations in Rubella Immunity

What is the rate of rubella immunity in the world? Natural immunity worldwide is estimated at 80–85% (Bantavala and Brown, 2004; Plotkin, 2006). With wide vaccine coverage, areas of Europe, Australia, and Turkey estimate 85–90% of their populations are immune (Madi et al., 2014). Not differentiating natural immunity versus vaccine induced, global immunity to rubella ranges from 95% in Kuwait, 77.5% in Russia, 76% in Sri Lanka, 71.1% in Vietnam, 54.1% in Nigeria to 40% in Panama (Jacobson et al., 2009; Madi et al., 2014; Miyakawa et al., 2014). More recently, Kuwait estimates only 88.4% of their population is immune. Of the 11.6% who are non-immune, 4% are Kuwaiti citizens with wide vaccine coverage, and 7.6% are immigrant workers with inconsistent vaccine coverage (Madi et al., 2014). In addition to waning titers and the influx of unvaccinated immigrants, rubella non-immunity may be increasing due to those concerned about MMR vaccines causing autism opting for their children not to be vaccinated. In the UK, a surveillance study has shown the rate of rubella non-immunity increasing from 4.1 to 6.8% over 5 years (Mortlock and Farthing, 2014). The rate of autism in the UK is 1 in 102 (Shuman, 2014). In Japan, vaccine gaps have occurred with regards to measles, mumps, and rubella. Of those who opted out of vaccination, 12.4% are non-immune to rubella (Okuda et al., 2008). Japan has had recent rubella outbreaks with the outbreak in 2012–2013 causing 40 cases of CRS as of March 2014 (Ohkusa et al., 2014; Saitoh and Okabe, 2014). Of pregnant women in Japan, 1.1% test positive for rubella-specific IgM (Okuda et al., 2008). Incidentally, the rate of autism in Japan is one of the highest in the world, 1 in 55 (Shuman, 2014).

Waning titers are common in populations with vaccine schedules to rubella, but what happens in populations without such vaccine schedules? In Vietnam, a prospective surveillance study done in 2014 diagnosed 113 cases of CRS, giving a rate of 1.13 per 1000 births in Hanoi (VanBang et al., 2014). A similar 2014 study conducted in Nha Trang, Vietnam, found a CRI rate of 1.51/1000 live births, and that older moms were more likely to have natural immunity (Miyakawa et al., 2014). A 2014 paper from Tanzania, where rubella vaccines are not scheduled, estimated natural immunity in pregnant women at 92.6%. In older women, ages 25–44 years, 94% had natural immunity. In younger women, ages 15–24 years, 89% had natural immunity. The authors estimate natural immunity increases each year by 12%, and that the high natural rate of immunity suggests ongoing endemic transmission. They also tested pregnant women for rubella-specific IgM and found 0.3% to be positive in Tanzania (Mwambe et al., 2014). In the developing world, older moms have more natural immunity. Contrasting what occurs in the developed world with more vaccine coverage, older moms have less immunity due to the decay of titers over time.

## Perinatal Risks of Autism and CRS, with Focus on the Foreign Born Mother

Many studies have looked at perinatal risk factors for autism; common among these risks are: prematurity, low APGARs, low or high birth weight, advanced maternal and paternal ages (AMA, APA), certain medicine exposures, vaginal bleeding in pregnancy, gestational diabetes, breech presentation, hyperbilirubinemia in the newborn, first born child, and foreign born mothers – especially if born in East Asia and Sub-Saharan Africa. One meta-analysis of such studies published in 2009, determined that AMA, APA, certain medicines, vaginal bleeding in pregnancy, gestational diabetes, and first born child all met statistical significance for risk factors of autism. Mother born abroad had an increase of 28% with a *p*-value of 0.006 (Gardener et al., 2009). Another meta-analysis published in 2012, found that AMA, APA, first born child, medicines, foreign born mother, small for gestational age, hyperbilirubinemia, breech presentation, preterm delivery, and low APGARs all met significance for risk factors of autistic spectrum, including pervasive developmental disorder (Guinchat et al., 2012). Sweden has commonly cited that foreign born mothers have an increased risk of having a child with autism, with East Asian moms having an OR of 3.4, and Sub-Saharan African mothers having an OR of 7.3 (Haglund and Kallen, 2011). Further Swedish studies have shown that Somali born mothers had a three to four times higher risk of having children with autism in 1988–1999, and then this increased to a four to five times higher risk in 1999–2003. In the latter estimate, the risk of a Somali woman giving birth to a child with autism was 0.98 versus 0.21% in a group not of Somali background. Also >80% of autistic children born to Somali women had “profound hyperactivity” (Barnevik-Olsson et al., 2010). The Swedes have also calculated that 15% of children born to mothers from Uganda had autism, 200 times that of their general population (Gillberg et al., 1995). How could these risk factors fit with CRI? Elevated bilirubin and low birth weight are lesser signs seen with CRS/CRI. APA and AMA correlate with waning immunity or increased susceptibility to rubella in developed countries. First born children may be more affected because once a mother becomes naturally immune, she better resists subsequent infection. Foreign born mothers (from the developing world, without vaccinations) are less likely to be immune or again more likely to be susceptible to rubella.

Similar to cases of autism, many cases of CRS in developed nations are born to women originally from areas without rubella vaccination programs, especially Sub-Saharan Africa, Asia and South America (Bantavala and Brown, 2004; Banerji et al., 2005). In the US, from 2004 to 2012, five of six cases of CRS occurred in children born to mothers either originating from or having recently traveled to Tanzania, Nigeria, Sudan, Ivory Coast, and Asia (with travel to India, China, and Singapore) (Jacobson et al., 2009; CDC, 2013). In Australia, from 2004 to 2013, of five cases of CRS, all five of the children’s mothers were born overseas, including Indonesia, India, Nepal, and Thailand (Khandaker et al., 2014).

## The Global Circulation of Rubella

Developed nations are not the only countries to see imported rubella. Rubella cases increase in areas of greater population density, and different strains circulate with the increased international movement of people (Plotkin, 2006; Yoshikura, 2014). In Tunisia, all known outbreaks of rubella prior to 2011 had been of a strain indigenous to Africa, but during the 2011–2012 outbreak, an imported strain was isolated in several Tunisian cases. With this outbreak, there was a large incidence of neurological symptoms, with 39 of the 280 confirmed cases having encephalitis (Messedi et al., 2014). Certain circulating strains today might have more neurotropism. From 2007 to 2011, Taiwan has been following five rubella strains of imported “clusters,” from Vietnam, Malaysia, Thailand, Germany, and South Africa (Cheng et al., 2013). In Brazil, they have recently isolated four genotypes of rubella (Curti et al., 2013). In Europe rubella cases are on the rise with 29,601 cases in 2012, and 39,367 cases in 2013. They estimate an increase in 10,000 cases per year, claiming much of the cases occur in Poland (Muscat et al., 2014). In Poland, a 2011–2012 study states that only 0.2% of cases are diagnosed by serology, and 99.8% are clinically diagnosed. If 50–75% of cases are subclinical, then estimates of rubella cases are assuredly too low (Rogalska, 2014). As cities are more populated than ever, and people trot the globe at ever increasing paces, rubella thrives. Rubella has global distribution and will be impossible to eliminate without extensive, repetitive, and improved vaccination on a global scale.

## Autism and Vaccination

If rubella is a current cause of autism, then why does autism exist in vaccinated populations? As stated previously, vaccination does not prevent reinfection. Primary failures occur when the rubella vaccine fails to elicit protective antibodies. Secondary failures also occur, when persons with immunity to rubella are reinfected. Possibly some of autism is what CRS has become today; CRS with less morbidity and mortality at birth, but more development of behavioral and neurological effects. If rubella infects 1 in 1000 to 1 in 100,000 cells in the fetus of a rubella naïve individual (Webster, 1998), perhaps it infects an even lesser ratio in an individual with incomplete rubella immunity. Inflammation in the latter individual would build quickly enough to limit viral spread. Rubella is much more likely to come in contact with a partially immune host today than decades ago when CRS was first described.

Proponents who claim that the MMR vaccine causes autism have forced studies to compare the prevalence of autism in vaccinated and unvaccinated populations. Studies looking at vaccinated versus unvaccinated populations might be expected to show a decreased prevalence of autism in vaccinated groups, but unfortunately vaccinated and unvaccinated groups do not equate with immunity and non-immunity to rubella. In reality, vaccinated groups have an estimated 86–97% immunity (Jacobson et al., 2009), and unvaccinated groups have an estimated 80–85% natural immunity (Plotkin, 2006; Nguyen et al., 2013). To blur the picture a bit more, those with natural immunity resist reinfection with rubella better than those with vaccine-induced immunity. Even if rubella causes autism, these studies may not find much difference between the two groups. Further, the aim of these studies is not to see if rubella causes autism, but to see if the MMR vaccine causes autism. Overwhelmingly, they show that the MMR vaccine does not cause autism as there is no statistically significant increase in diagnoses of autism in the vaccinated group. In fact most studies, including a meta-analysis and a large Danish population study, show less diagnoses of autism in the vaccinated groups. The large meta-analysis involved five cohorts consisting of >1.2 million children, showing a decreased odds ratio of 0.84 for autism in the MMR vaccination group (Taylor et al., 2014). The Danish population study of 537,303 children, 440,665 receiving MMR vaccination, had a decreased relative ratio of 0.92 for autism and 0.83 for ASD (Madsen et al., 2002). In Japan, the MMR vaccine was withdrawn in 1992, and a substantial increase of autism was seen, rising “dramatically,” with the birth cohort of 1993. Japan’s incidence of autism increased from approximately 0.6% in 1992 to 1.6% in 1994 (Honda et al., 2005). Withdrawing the MMR vaccine did not decrease the incidence of autism, but may have inadvertently increased the incidence of autism, perhaps by increasing the susceptibility to rubella. In Poland, an interesting case-control study of 96 individuals with autism and 192 matched controls without autism showed that autism in the MMR group versus the unvaccinated group had an OR of 0.17; autism in the measles only vaccine group versus the unvaccinated group had an OR of 0.44; and autism in the MMR group versus the measles only vaccine group had an OR of 0.47 (Mrozek-Budzyn et al., 2010). Though small, this study suggests that a non-measles component of the MMR vaccine might be associated with less autism. Other MMR studies do not stratify a measles only vaccine arm. In summary, studies of MMR vaccinated and non-vaccinated groups do not show an increase of autism in their vaccinated arms, and most trend a decrease in the vaccinated arms.

## CRS and CRI Today

If rubella is a cause of autism, then why is autism so prevalent, but CRS is not? First rubella likely causes a certain, yet to be determined, percentage of autism, but not all autism. Second, rubella can cause spontaneous abortions, and prenatal diagnoses of rubella can lead to elective abortions. In the 1964 rubella epidemic, there were 20,000 cases of CRS and 11,250 abortions, though these abortions are not further classified as spontaneous or elective (Jacobson et al., 2009). The paper by Miller, who followed 1000 mothers with documented rubella infections in various stages of pregnancy, states that pregnancy continued in 40% of women; terminations (mostly induced) occurred in 60% (Miller et al., 1982). More recently (1992) in Pakistan, of 350 pregnancies, rubella-specific IgM was positive in 5% of the 143 mothers who carried to a live birth, and positive in 18% of the 212 mothers who naturally aborted. They speculated that rubella is a significant cause of miscarriage in Pakistan (Ahmed, 1992). A 2014 review surveyed 31 studies from developed countries, each study reporting outcomes of at least 30 maternal rubella infections. This review of almost 100 prenatal cases of CRI from 1999 to 2005 showed that prenatal diagnoses in the developed world often led to elective terminations (Thompson et al., 2014). A third reason CRS infants are not common has to do with reporting. In South Korea, three cases of CRS were reported to the government from 2001 to 2009 (9 years), but investigating insurance claims, 76 cases of CRS were reported to insurance companies from 2005 to 2009 (5 years) (Choe et al., 2010). In a region of Italy, no cases of CRS were reported to the regional government, but pulling hospital records revealed one case of CRS, two laboratory confirmed cases of CRI, four suspected cases of CRI, and seven cases of rubella in pregnancy (Cozza et al., 2015). In 2006, the WHO estimated at least 100,000 cases of CRS worldwide (Robinson et al., 2006); this can only be estimated as most cases occur in areas with little reporting, but even in areas with reporting, cases are frequently not counted.

Given the WHO estimate of 100,000 cases of CRS in the world; what might then be the incidence of CRI? Or rather how many pregnancies are affected by rubella? Worldwide this estimate is hard to guess. If only clinically diagnosed cases are considered, then 66–75% of CRI cases will be missed and lack follow-up. After the 1964 rubella epidemic in the US, a prospective study followed 4005 pregnancies with CRI, 68% of the CRI infants were asymptomatic at birth; but following these seemingly normal children, 71% developed manifestations (mostly hearing loss and intellectual disabilities) by 5 years of age (Dobson, 2014). That was 50 years ago; what about data today? There is no reason to check umbilical cord blood for rubella as was done in the 60s and 70s, and babies born with to mothers with prior vaccinations and subclinical rubella infections, likely will not teem with blueberry muffin spots. Still, a few studies have measured rubella-specific IgM in pregnant mothers, finding 5% positive in Pakistan (Ahmed, 1992), 1.1% positive in Japan (Okuda et al., 2008), 0.3% positive in Tanzania (Mwambe et al., 2014), estimating that rubella affects somewhere between 0 and 5% of pregnancies globally. A recent study from Nigeria tested 300 pregnant women for rubella-specific IgG, finding 53% positive for IgG. Only those 53% positive for IgG were further tested for IgM, finding 10% positive for rubella-specific IgM. The percentage of the initial group positive for IgM is unknown as only those positive for IgG were tested for IgM, but is at least 5.3% (Onakewhor and Chiwuzie, 2011). In the US, a study in Houston, of 298 women who tested rubella non-immune early in pregnancy, 19 naturally converted to immune at the time of delivery by at least a fourfold increase in titer; 6.38% of women initially non-immune to rubella became immune to rubella over a range of just 10–36 weeks. None of the women had any known exposures or symptoms (Hutton et al., 2014). The title of the Houston paper hints at a reemergence of rubella, but more likely the study confirms rubella’s persistence. In these last five papers (Ahmed, 1992; Okuda et al., 2008; Onakewhor and Chiwuzie, 2011; Hutton et al., 2014; Mwambe et al., 2014), only serology estimated the prevalence of rubella, without serology, doubtfully any presence of rubella would be known. Rubella is still “quietly getting away with it,” but to what extent can only be guessed.

What is the true burden of CRI? Is it autism? And if so, how much autism is caused by rubella virus? An older, frequently quoted study by Fombonne revealed 0.6% of a group of 174 autistic individuals also had a diagnosis of CRS (Fombonne et al., 1997). Another study, using Chess’s initial prevalence of 7.4%, an average birth rate of 4,150,000 per year for the years 2001–2010 in the US, and a pre-vaccination rate of CRS of 4/10,000, calculated (by multiplying 0.074, the birth rate and 4/10,000) 1228 cases of autism were prevented each year in the US from 2001 to 2010 by vaccination to rubella (Berger et al., 2011). But this estimate assumes that there are no current CRI/CRS cases in the US, and that MMR vaccination is 100% effective. The thought that vaccination has wiped out rubella is falsely reassuring and has managed to wipe out most rubella research, but unfortunately rubella lingers. In summary, these estimates are probably too exclusive, missing the subclinical CRI in vaccinated mothers with immune systems partially primed to mount an inflammatory response with cascading changes in the fetal brain. Other facts to consider are the increasing rubella cases with circulation from continent to continent, the continual rubella susceptibility as vaccines are not given globally and are not fully protective, and the exposure of rubella in 0–5% of pregnancies worldwide. But of pregnancies affected by rubella, how many offspring have autism? Is it consistent with the estimates of Chess and Desmond in the 60s–70s: 7.41 (Chess, 1971), 10.24 (Chess, 1977), or 12.5% (Desmond et al., 1969); or is it much higher now that the diagnosis of autism has expanded to be so much more inclusive?

## Conclusion

Rubella, once widespread and widely studied 30–50 years ago, was linked to autism. Chess proposed that CRS children have 200 times the prevalence of autism at a time when the diagnosis of autism was much more limited. Although rubella is one of many maternal infections with possible links to autism, no other infection boasts such an increased prevalence; perhaps it is time to give rubella another look. Autism shares some overlap with the three most common findings in babies with CRS: deafness, CHD, and to a lesser extent visual changes. Both CRS children and autistic children can have hyperactivity, spasticity, and may develop DM1 as young adults. Neuropathology and neuroradiology of children with both CRS and ASD often show brains, which are normal or with hints of a faint viral signature of periventricular calcifications and deep white matter or temporal lobe changes; this possibly owing to rubella’s sporadic nature, infecting infrequent foci of cells and inhibiting their mitosis, maturation, migration, and connections. Rubella affects brain arterioles leading to decreased perfusion in certain areas of the brain. PET scans of children with autism show decreased perfusion in similar areas of the brain. In both CRS and autism, changes in the brain implicate the immune system. These findings parallel each other even though the bulk of CRS research is from 40 years ago, and the bulk of autism research is from the past decade. CRS children lack antibodies to rubella in 10–20% of cases, as do autistic children. Trying to map out the genetics of autism has been quite divergent, though one converging theory could be a host susceptibility to rubella regardless of vaccination. This susceptibility to rubella helps to explain rubella’s persistence, even in widely vaccinated populations. Genetics does not explain why in certain areas of the world both CRS and autistic cases are seemingly imported, or why rubella and autism cross many ethnicities with a global distribution. Though rubella research has diminished in the last 20 years, current evidence linking autism to rubella still exists.

## Going Forward

Research must develop to see if rubella plays a role in today’s cases of autism. If such studies demonstrated the continued existence of rubella, further studies, steps, and measures would be necessary to protect mothers and infants.

Possible studies might include

- As in the Houston study listed above, of mothers who convert from non-immune early in pregnancy to immune at the end of pregnancy (Hutton et al., 2014), their resulting children could be followed for development of autism and or behavioral outcomes.
- Pregnant women presenting with flu, cold, or viral symptoms could be tested for rubella.
- The Finnish study found 1.1% of children had evidence of rubella immunity before ever receiving MMR (Davidkin et al., 2008). Children could be similarly checked for rubella immunity before vaccination, and those demonstrating natural immunity could be monitored for development of autism. One caveat would be that certain children exposed *in utero* might not make antibodies.
- Stored umbilical cord blood could be tested for rubella.
- Blinded radiologists could read MRI and PET studies of known cases of CRI and autism for inter-rater and intra-rater reliability.
- Children with regressive autism could be evaluated for any evidence of rubella infection.
- Brain pathology specimens from autistic individuals could be investigated for rubella, especially in areas of heterotopic tissue.

## Author Contributions

The author confirms being the sole contributor of this work and approved it for publication.

## Conflict of Interest Statement

The author declares that the research was conducted in the absence of any commercial or financial relationships that could be construed as a potential conflict of interest.

## References

[B1] Aboudy Y. Barnea B. Yosef L. Frank T. Mendelson E. (2000). Clinical rubella reinfection during pregnancy in a previously vaccinated woman. J. Infect. 41, 187–189. 10.1053/jinf.2000.0716 11023770

[B2] Acosta M. T. Pearl P. L. (2003). The neurobiology of autism: new pieces of the puzzle. Curr. Neurol. Neurosci. Rep. 3, 149–156. 10.1007/s11910-003-0067-0 12583844

[B3] Adamo M. P. Zapata M. Frey T. K. (2008). Analysis of gene expression in fetal and adult cells infected with rubella virus. Virology 370, 1–11. 10.1016/j.virol.2007.08.003 17920097 PMC2694049

[B4] Ahmed M. U. (1992). IgM and IgG antibodies specific to rubella in child bearing women. J. Pak. Med. Assoc. 42, 121–122. 1507389

[B5] Ashwood P. Krakowiak P. Hertz-Picciotto I. Hansen R. Pessah I. Van deWater J. (2011). Elevated plasma cytokines in autism spectrum disorders provide evidence of immune dysfunction and are associated with impaired behavioral outcomes. Brain Behav. Immun. 25, 40–45. 10.1016/j.bbi.2010.08.003 20705131 PMC2991432

[B6] Aycock W. L. Ingalls T. H. (1946). Maternal disease as a principal in the epidemiology of congenital anomalies; with a review of rubella. Am. J. Med. Sci. 212, 336–379. 10.1097/00000441-194609000-00016 20996985

[B7] Bailey A. LeCouteur A. Gottesman I. Bolton P. Simonoff E. Yuzda E. (1995). Autism as a strongly genetic disorder: evidence form a British twin study. Psychol. Med. 25, 63–77. 10.1017/S0033291700028099 7792363

[B8] Bailey A. Luthert P. Dean A. Harding B. Janota I. Montgomery M. (1998). A clinicopathological study of autism. Brain 121, 889–905. 10.1093/brain/121.5.889 9619192

[B9] Banerji A. Ford-Jones E. L. Kelly E. Robinson J. L. (2005). Congenital rubella syndrome despite maternal antibodies. CMAJ 172, 1678–1679. 10.1503/cmaj.050230 15967967 PMC1150255

[B10] Bantavala J. E. Brown D. W. G. (2004). Rubella. Lancet 363, 1127–1137. 10.1016/S0140-6736(04)15897-2 15064032

[B11] Barnevik-Olsson M. Gillberg C. Fernell E. (2010). Prevalence of autism in children of Somali origin living in Stockholm: brief report of an at-risk population. Dev. Med. Child Neurol. 52, 1167–1168. 10.1111/j.1469-8749.2010.03812.x 20964674

[B12] Berger B. E. Navar-Boggan M. Omer S. B. (2011). Congenital rubella syndrome and autism spectrum disorder prevented by rubella vaccination – United States, 2001-2010. BMC Public Health 11:340 10.1186/1471-2458-11-340 21592401 PMC3123590

[B13] Best J. M. Banatvala J. E. Morgan-Capner P. Miller E. (1989). Fetal infection after maternal reinfection with rubella: criteria for defining reinfection. Br. Med. J. 299, 773–775. 10.1136/bmj.299.6702.773 2508917 PMC1837646

[B14] Boddaert N. Zilbovicius M. Philipe A. Robel L. Bourgeois M. Barthelemy C. (2009). MRI findings in 77 children with non-syndromic autistic disorder. PLoS ONE 4:e4415. 10.1371/journal.pone.0004415 19204795 PMC2635956

[B15] Brunelle F. Boddaert N. Zilbovicius M. (2009). Autism and brain imaging. Bull. Acad. Natl. Med. 193, 287–297. 19718886

[B16] Bullens D. Smets K. Vanhaesebrouck P. (2000). Congenital rubella syndrome after maternal reinfection. Clin. Pediatr. 39, 113–116. 10.1007/BF01710747 10696549

[B17] Carlsson L. H. Norrelgen F. Kjellmer L. Westerlund J. Gillberg C. Fernell E. (2013). Coexisting disorders and problems in preschool children with autism spectrum disorders. ScientificWorldJournal 2013, 213979. 10.1155/2013/213979 23737708 PMC3655672

[B18] CDC. (2013). Three cases of congenital rubella syndrome in the postelimination era – Maryland, Alabama, and Illinois, 2012. MMWR Morb. Mortal. Wkly. Rep. 62, 226–229. 10.1097/INF.0b013e318296b08a 23535689 PMC4605004

[B19] Cheng W. Y. Wang H. C. Liu M. T. Wu H. S. (2013). Molecular surveillance of rubella viruses in Taiwan from 2005 to 2011. J. Med. Virol. 85, 745–753. 10.1002/jmv.23451 23417619

[B20] Chess S. (1971). Autism in children with congenital rubella. J. Autism Child. Schizophr. 1, 33–47. 10.1007/BF01537741 5172438

[B21] Chess S. (1977). Follow-up report on autism in congenital rubella. J. Autism Child. Schizophr. 7, 69–74. 10.1007/BF01531116 576606

[B22] Choe Y. J. Lee S. T. Song K. M. Cho H. Bae G. R. Lee J. K. (2010). Surveillance and control of rubella in the republic of Korea from 2001 to 2009: the necessity for enhanced surveillance to monitor congenital rubella syndrome. Osong Public Health Res. Perspect. 1, 23–28. 10.1016/j.phrp.2010.12.007 24159436 PMC3766894

[B23] Christensen D. VanNaardenBraun K. Doernberg N. S. Maenner M. J. Arneson C. L. Durkin M. S. (2014). Prevalence of cerebral palsy, co-occurring autism spectrum disorders, and motor functioning – autism and developmental disabilities monitoring network, USA 2008. Dev. Med. Child Neurol. 56, 59–65. 10.1111/dmcn.12268 24117446 PMC4351771

[B24] Christenson B. Bottiger M. (1994). Long-term follow-up study of rubella antibodies in naturally immune and vaccinated young adults. Vaccine 12, 41–45. 10.1016/0264-410X(94)90009-4 8303939

[B25] Coyle P. K. Wolinsky J. S. Buimovici-Klein E. Moucha R. Cooper L. Z. (1982). Rubella-specific immune complexes after congenital infection and vaccination. Infect. Immun. 36, 498–503. 7085069 10.1128/iai.36.2.498-503.1982 PMC351255

[B26] Cozza V. Martinelli D. Cappelli M. G. Fortunato F. Prato R. (2015). Further efforts in the achievement of congenital rubella syndrome/rubella elimination. Hum. Vaccin. Immunother. 11, 220–224. 10.4161/hv.36154 25483539 PMC4514341

[B27] Curti S. P. Figueiredo C. A. deOliveira M. I. Andrade J. Q. Zugaib M. Yu A. L. F. (2013). Molecular epidemiology of rubella viruses involved in congenital rubella infections in Sao Paulo, Brazil, between 1996 and 2009. J. Med. Virol. 85, 2034–2041. 10.1002/jmv.23675 23861141 PMC7167121

[B28] Davidkin I. Jokinen S. Broman M. Leinikki P. Peltola H. (2008). Persistence of measles, mumps, and rubella antibodies in an MMR-vaccinated cohort: a 20-year follow-up. J. Infect. Dis. 197, 950–956. 10.1086/528993 18419470

[B29] Desmond M. M. Montgomery J. R. Melnick J. L. Cochane G. G. Verniaud W. (1969). Congenital rubella encephalitis. Effects on growth and early development. Am. J. Dis. Child. 118, 30–31. 10.1001/archpedi.1969.02100040032005 5815336

[B30] Dilber C. Calışkan M. Sönmezoğlu K. Nişli S. Mukaddes N. M. Tatlı B. (2013). Positron emission tomography findings in children with infantile spasms and autism. J. Clin. Neurosci. 20, 373–376. 10.1016/j.jocn.2012.03.034 23219829

[B31] Dobson S. R. (2014). Congenital Rubella Syndrome: Clinical Features and Diagnosis. Available at: http://www.uptodate.com/contents/congenital-rubella-syndrome-clinical-featues-and-diagnosis

[B32] Dunn P. M. (2007). Perinatal lessons from the past: Sir Norman Gregg, ChM, MC, of Sydney (1892-1966) and rubella embryopathy. Arch. Dis. Child. Fetal Neonatal Ed. 92, F513–F514. 10.1136/adc.2005.091405 17951553 PMC2675410

[B33] Enders G. Calm A. Schaub J. (1984). Rubella embryopathy after previous maternal rubella vaccination. Infection 12, 96–98. 10.1007/BF01641680 6735484

[B34] Fombonne E. DuMazaubrun C. Cans C. Grandjean H. (1997). Autism and associated medical disorders in a French epidemiological survey. J. Am. Acad. Child Adolesc. Psychiatry 36, 1561–1569. 10.1097/00004583-199711000-00019 9394941

[B35] Gardener H. Spiegelman D. Buka S. L. (2009). Prenatal risk factors for autism: a comprehensive meta-analysis. Br. J. Psychiatry 195, 7–14. 10.1192/bjp.bp.108.051672 19567888 PMC3712619

[B36] Gentile I. Bravaccio C. Bonavolta R. Zappulo E. Scarica S. Riccio M. P. (2013). Response to measles-mumps-rubella vaccine in children with autism spectrum disorders. In Vivo 27, 377–382. 23606694

[B37] Gillberg C. Schaumann H. Gilberg I. C. (1995). Autism in immigrants: children born in Sweden to mothers born in Uganda. J. Intellect. Disabil. Res. 39(Pt2), 141–144. 10.1111/j.1365-2788.1995.tb00482.x 7787384

[B38] Goldani A. S. Downs S. R. Widjaja F. Lawton B. Hendren R. L. (2014). Biomarkers in autism. Front. Psychiatry 5:100. 10.3389/fpsyt.2014.00100 25161627 PMC4129499

[B39] Gregg N. M. (1991). Congenital cataract following German measles in the mother. 1941. Epidemiol. Infect. 107, 3–14. 10.1017/S0950268800048627 PMC2272051 1879476

[B40] Guinchat V. Thorsen P. Laurent C. Cans C. Bodeau N. Cohen D. (2012). Pre-, peri- and neonatal risk ractors for autism. Acta Obstet. Gynecol. Scand. 91, 287–300. 10.1111/j.1600-0412.2011.01325.x 22085436

[B41] Haglund N. G. Kallen K. B. (2011). Risk factors for autism and Asperger syndrome. Perinatal factors and migration. Autism 15, 163–183. 10.1177/1362361309353614 20923887

[B42] Harcourt G. C. Best J. M. Banatvala J. E. (1980). Rubella-specific serum and nasopharyngeal antibodies in volunteers with naturally aquired and vaccine-induced immunity after intranasal challenge. J. Infect. Dis. 142, 145–155. 10.1093/infdis/142.2.145 7410897

[B43] Hashimoto T. Sasaki M. Fukumizu M. Hanaoka S. Sugai K. Matsuda H. (2000). Single-photon emission computed tomography of the brain in autism: effect of the development level. Pediatr. Neurol. 23, 416–420. 10.1016/S0887-8994(00)00224-1 11118797

[B44] Hitglou M. Ververi A. Antoniadis A. Zafeiriou D. I. (2010). Childhood autism and auditory system abnormalities. Pediatr. Neurol. 42, 309–314. 10.1016/j.pediatrneurol.2009.10.009 20399382

[B45] Honda H. Shimizu Y. Rutter M. (2005). No effect of MMR withdrawal on the incidence of autism: a total population study. J. Child Psychol. Psychiatry 46, 572–579. 10.1111/j.1469-7610.2005.01425.x 15877763

[B46] Horstmann D. M. Liebhaber H. LeBouvier G. Rosenberg D. A. Halstead S. B. (1970). Rubella: reinfection of vaccinated and naturally immune persons exposed in an epidemic. N. Engl. J. Med. 283, 771–778. 10.1056/NEJM197010082831501 5456233

[B47] Hutton J. Hutton G. J. (2014). Congenital rubella with autism and evidence of a remote stroke. J. Vaccines Vaccin. 5, 258 10.4172/2157-7560.1000258

[B48] Hutton J. Rowan P. Greisinger A. Mouzoon M. (2014). Rubella monitoring in pregnancy as a means for evaluating a possible re-emergence of rubella. Am. J. Obstet. Gynecol. 211, 534 10.1016/j.ajog.2014.05.046 24887317

[B49] Hyde T. B. Sato H. K. Hao L. Flannery B. Zheng Q. Wannemuehler K. (2014). Identification of serologic markers for school-aged children with congenital rubella syndrome. J. Infect. Dis. 212, 57–66. 10.1093/infdis/jiu604 25362195 PMC4654588

[B50] Jacobson R. M. OvsyannikovaI G. Poland G. A. (2009). Genetic basis for variation of vaccine response: our studies with rubella vaccine. Paediatr. Child Health 19(Suppl. 2), S156–S159. 10.1016/j.paed.2009.08.019 20976024 PMC2957833

[B51] Kabatas E. U. Ozzer P. A. Ertugrul G. T. Kurtul B. E. Bodur S. Alan B. E. (2015). Initial opthalmic findings in Turkish children with autistic spectrum disorder. J. Autism Dev. Disord. 45, 2578–2581. 10.1007/s10803-015-2428-3 25800865

[B52] Ke X. Tang T. Hong S. Hang Y. Zou B. Li H. (2009). White matter impairments in autism, evidence from voxel-based morphometry and diffusion tensor imaging. Brain Res. 1265, 171–177. 10.1016/j.brainres.2009.02.013 19233148

[B53] Kennedy R. B. OvsyannikovaI G. HaralambievaI H. Lambert N. D. Pankratz V. S. Poland G. A. (2014). Genetic polymorphisms associated with rubella virus-specific cellular immunity following MMR vaccination. Hum. Genet. 133, 1407–1417. 10.1007/s00439-014-1471-z 25098560 PMC4249927

[B54] Khandaker G. Zurynski Y. Jones C. (2014). Surveillance for congenital rubella in Australia since 1993: cases reported between 2004 and 2013. Vaccine 32, 6746–6751. 10.1016/j.vaccine.2014.10.021 25454858

[B55] Kohane I. S. McMurry A. Weber G. MacFadden D. Rappaport L. Kunkel L. (2012). The co-morbidity burden of children and young adults with autism spectrum disorders. PLoS ONE 7:e33224. 10.1371/journal.pone.0033224 22511918 PMC3325235

[B56] Kouri G. Aguilera A. Rodriguez P. Korolev M. (1974). A study of microfoci and inclusion bodies produced by rubella virus in the RK-13 cell line. J. Gen. Virol. 22, 73–80. 10.1099/0022-1317-22-1-73 4591671

[B57] Kremer J. R. Schneider F. Muller C. P. (2006). Waning antibodies in measles and rubella vaccinees – a longitudinal study. Vaccine 24, 2594–2601. 10.1016/j.vaccine.2005.12.015 16427163

[B58] Kremerrr J. R. Muller C. P. (2005). Evaluation of commercial assay detecting specific immunoglobulin g in oral fluid for determining measles immunity in vaccinees. Clin. Diagn. Lab. Immunol. 12, 668–670. 10.1128/CDLI.12.5.668-670.2005 15879031 PMC1112091

[B59] Kuroda Y. Matsui M. (1997). Progressive rubella panencephalitis. Nihon Rinsho 55, 922–925. 9103895

[B60] Lane B. Sullivan E. V. Lim K. O. Beal D. M. Harvey R. L. Jr. Meyers T. (1996). White matter MR hyperintensities in adult patients with congenital rubella. AJNR Am. J. Neuroradiol. 17, 99–103. 8770257 PMC8337970

[B61] Lee J. Y. Bowden D. S. (2000). Rubella virus replication and links to teratogenicity. Clin. Microbiol. Rev. 13, 571–587. 10.1128/CMR.13.4.571-587.2000 11023958 PMC88950

[B62] Libbey J. E. Coon H. H. Kirkman N. J. Sweeten T. L. Miller J. N. Lainhart J. E. (2007). Are there altered antibody responses to measles, mumps, or rubella viruses in autism? J. Neurovirol. 13, 252–259. 10.1080/13550280701278462 17613715

[B63] Lin C. C. Yang C. Y. Shih Y. L. Huang Y. Y. Yang T. H. Liang J. Y. (2012). Persistence and titer changes of rubella virus antibodies in primiparous women who had been vaccinated with strain RA 27/3 in junior high school. Clin. Vaccine Immunol. 1, 1–4. 10.1128/CVI.05334-11 22072722 PMC3255950

[B64] Lindquist J. M. Plotkin S. A. Shaw L. Gilden R. V. Williams M. L. (1965). Congenital rubella syndrome as a systemic infection. Studies of affected infants born in Philadelphia, U.S.A. Br. Med. J. 2, 1401–1406. 10.1136/bmj.2.5475.1401 5892115 PMC1847241

[B65] Lintas C. Altieri L. Lombardi F. Sacco R. Persico A. M. (2010). Association of autism with polyomavirus infection in postmortem brains. J. Neurovirol. 16, 141–149. 10.3109/13550281003685839 20345322

[B66] Madi N. Al-Tawalah H. Khalik D. A. Al-Nakib W. (2014). A relatively high number of pregnant women in Kuwait remain susceptible to rubella: a need for an alternative vaccination policy. Med. Princ. Pract. 23, 145–148. 10.1159/000356892 24434233 PMC5586858

[B67] Madsen K. M. Hviid A. Vestergaard M. Schendel D. Wohlfahrt J. Thorsen P. (2002). A population-based study of measles, mumps, and rubella vaccination and autism. N. Engl. J. Med. 347, 1477–1482. 10.1056/NEJMoa021134 12421889

[B68] Mauracher C. A. Mitchell L. A. Tingle A. J. (1993). Selective tolerance to the E1 protein of rubella virus in congenital rubella syndrome. J. Immunol. 151, 2041–2049. 8345195

[B69] McFadden K. Minshew N. J. (2013). Evidence of dysregulation of axonal growth and guidance in the etiology of ASD. Front. Hum. Neurosci. 7:671 10.3389/fnhum.2013.00671 24155705 PMC3804918

[B70] Messedi E. Fki-Berrajah L. Gargouri S. Chouikha A. Chaari A. Bouaziz M. (2014). Clinical epidemiological and molecular aspects of rubella outbreak with high number of neurological cases, Tunisia 2011-2012. J. Clin. Virol. 61, 248–254. 10.1016/j.jcv.2014.07.003 25088766

[B71] Michel M. Schmidt M. J. Mirnics K. (2012). Immune system gene dysregulation in autism and schizophrenia. Dev. Neurobiol. 72, 1277–1287. 10.1002/dneu.22044 22753382 PMC3435446

[B72] Miller E. Cradock-Watson J. E. Pollock T. M. (1982). Consequences of confirmed maternal rubella at successive stages of pregnancy. Lancet 2, 781–784. 10.1016/S0140-6736(82)92677-0 6126663

[B73] Miron D. On A. (1992). Congenital rubella syndrome after maternal immunization. Harefuah 122, 291–293. 1572573

[B74] Miyakawa M. Yoshino H. Yoshida L. M. Vynnycky E. Motomura H. Tho le H. (2014). Seroprevalence of rubella in the cord of pregnant women and congenital rubella incidence in Nha Trang, Vietnam. Vaccine 32, 1192–1198. 10.1016/j.vaccine.2013.08.076 24021315

[B75] Mortlock S. Farthing S. (2014). Rubella antibody status of patients attending a south-west London antenatal clinic, 2007-2012. Br. J. Biomed. Sci. 71, 111–114. 25265756 10.1080/09674845.2014.11669975

[B76] Mrozek-Budzyn D. Kieltyka A. Majewska R. (2010). Lack of association between measles-mumps-rubella vaccination and autism in children: a case-control study. Pediatr. Infect. Dis. J. 29, 387–400. 10.1097/INF.0b013e3181c40a8a 19952979

[B77] Muhle R. Trentacoste S. V. Rapin I. (2004). The genetics of autism. Pediatrics 113, e472–e486. 10.1542/peds.113.5.e472 15121991

[B78] Muscat M. Shefer A. BenMamou M. Spataru R. Jankovic D. Deshevoy S. (2014). The state of measles and rubella in the WHO European Region, 2013. Clin. Microbiol. Infect. 20(Suppl. 5), 12–18. 10.1111/1469-0691.12584 24520948

[B79] Mwambe B. Mirambo M. M. Mshana S. E. Massinde A. N. Kidenya B. R. Michael D. (2014). Sero-positivity rate of rubella and associated factors among pregnant women attending antenatal care in Mwanza, Tanzania. BMC Pregnancy Childbirth 1:95. 10.1186/1471-2393-14-95 24589180 PMC3975942

[B80] Nguyen T. V. Pham V. H. Abe K. (2013). Pathogenesis of congenital rubella virus infection in human fetuses: viral infection in the ciliary body could play an important role in cataractogenesis. J. Clin. Virol. 57, 59–63. 10.1016/j.ebiom.2014.10.021 26137534 PMC4484509

[B81] Numazaki K. Fujikawa T. (2003). Intracranial calcification with congenital rubella syndrome in a mother with serologic immunity. J. Child Neurol. 18, 296–297. 10.1177/08830738030180040601 12760434

[B82] Ohkusa Y. Sugawara T. Arai S. Satoh H. Okuno H. Tanakkka-Taya K. (2014). Short-term prediction of the incidence of congenital rubella syndrome. PLoS Curr. 6.10.1371%2Fcurrents.outbreaks.8c74272f4348781c5d01c81e6150c2f7 25642379 10.1371/currents.outbreaks.8c74272f4348781c5d01c81e6150c2f7 PMC4234431

[B83] Ohnishi T. Matsuda H. Hashimoto T. Kunihiro T. Nishikawa M. Uema T. (2000). Abnormal regional cerebral blood flow in childhood autism. Brain 123, 1838–1844. 10.1093/brain/123.9.1838 10960047

[B84] Okuda M. Yamanaka M. Takahashi T. Ishikawa H. Endoh M. Hirahara F. (2008). Positive rates for rubella antibody in pregnant women and benefit of post-partum vaccination in a Japanese perinatal center. J. Obstet. Gynaecol. Res. 34, 168–173. 10.1111/j.1447-0756.2007.00689.x 18412777

[B85] Onakewhor J. U. Chiwuzie J. (2011). Seroprevalence survey of rubella infection in pregnancy at the University of Benin Teaching Hospital, Benin City, Nigeria. Niger. J. Clin. Pract. 14, 140–145. 10.4103/1119-3077.84002 21860127

[B86] OvsyannikovaI G. Pankratz V. S. Larrabee B. R. Jacobson R. M. Poland G. A. (2014a). HLA genotypes and rubella vaccine immune response: additional evidence. Vaccine 32, 4206–4213. 10.1016/j.vaccine.2014.04.091 24837503 PMC4124933

[B87] OvsyannikovaI G. Salk H. M. Larrabee B. R. Pankratz V. S. Poland G. A. (2014b). Single-nucelotide polymorphism associations in common with immune responses to measles and rubella vaccines. Immunogenetics 66, 663–669. 10.1007/s00251-014-0796-z 25139337 PMC4198472

[B88] Pitts S. I. Wallace G. S. Montana B. Handschur E. F. Meislich D. Sampson A. C. (2014). Congenital rubella syndrome in child of woman without known risk factors, New Jersey, USA. Emerging Infect. Dis. 20, 307–309. 10.3201/eid2002.131233 24447409 PMC3901471

[B89] Plotkin S. A. (2006). The history of rubella and rubella vaccination leading to elimination. Clin. Infect. Dis. 43(Suppl. 3), S164–S168. 10.1086/505950 16998777

[B90] Razzag H. Oster M. Reefhuis J. (2015). Long-term outcomes in children with congenital heart disease: national health interview survey. J. Pediatr. 166, 119–124. 10.1016/j.jpeds.2014.09.006 25304924 PMC4378575

[B91] Robinson J. L. Lee B. E. Preiksaitis J. K. Plitt S. TipplesG A. (2006). Prevention of congenital rubella syndrome – what makes sense in 2006? Epidemiol. Rev. 28, 81–87. 10.1093/epirev/mxj007 16775038

[B92] Rogalska J. (2014). Rubella in Poland in 2012. Przegl. Epidemiol. 68, 195–199. 25135498

[B93] Rorke L. B. Spiro A. J. (1967). Cerebral lesions in congenital rubella syndrome. J. Pediatr. 70, 243–255. 10.1016/S0022-3476(67)80419-0 6018109

[B94] Saitoh A. Okabe N. (2014). Recent progress and concerns regarding the Japanese immunization program: addressing the “vaccine gap”. Vaccine 32, 4253–4258. 10.1016/j.vaccine.2014.06.022 24951864

[B95] Sawlani V. ShivaShankar J. J. White C. (2013). Magnetic resonance imaging findins in a case of congenital rubella encephalitis. Can. J. Infect. Dis. Med. Microbiol. 24, e122–e123. 10.1155/2013/858302 24489572 PMC3905013

[B96] Severino M. Zerem A. Biancheri R. R. Cristina E. Rossi A. (2014). Spontaneously regressing leukoencephalopathy with bilateral temporal cysts in congenital rubella infection. Pediatr. Infect. Dis. J. 33, 422–424. 10.1097/INF.0000000000000134 24153011

[B97] Sharma A. Gokulchandran N. Sane H. Nagrajan A. Paranjape A. Kulkarni P. (2013). Autologous bone marrow mononuclear cell therapy for autism: an open label proof of concept study. Stem Cells Int. 623875, 1–13. 10.1155/2013/623875 24062774 PMC3767048

[B98] Shi L. Smith S. E. P. Malkova N. Tse D. Su Y. Patterson P. H. (2009). Activation of the maternal immune system alters cerebellar development in the offspring. Brain Behav. Immun. 23, 116–123. 10.1016/j.bbi.2008.07.012 18755264 PMC2614890

[B99] Shuman L. (2014). The mystery of autism. MIT Technol. Rev. 118, 20.

[B100] Simmons D. R. Robertson A. E. McKay L. S. Toal E. McAleer P. Pollick F. E. (2009). Vision in autism spectrum disorders. Vision Res. 49, 2705–2739. 10.1016/j.visres.2009.08.005 19682485

[B101] Smits G. Mollema L. Hahné S. de Melker H. Tcherniaeva I. van der Klis F. (2014). Seroprevalence of rubella antibodies in the Netherlands after 32 years of high vaccination coverage. Vaccine 32, 1890–1895. 10.1016/j.vaccine.2014.01.066 24513012

[B102] Stubbs E. G. (1976). Autistic children exhibit undetectable hemagglutination-inhibition antibody titers despite previous rubella vaccination. J. Autism Child. Schizophr. 6, 269–274. 10.1007/BF01543467 1036494

[B103] Sundaram S. K. Kumar A. Makki M. I. Behen M. E. Chugani H. T. Chugani D. C. (2008). Diffusion tensor imaging of frontal lobe in autism spectrum disorder. Cereb. Cortex 18, 2659–2665. 10.1093/cercor/bhn031 18359780 PMC2567426

[B104] Taylor L. E. Swerdfeger A. L. Eslick G. D. (2014). Vaccines are not associated with autism: an evidence-based meta-analysis of case-control and cohort studies. Vaccine 32, 3623–3629. 10.1016/j.vaccine.2014.04.085 24814559

[B105] Thompson K. M. Simmons E. A. Badizadegan K. Reef S. E. Cooper L. Z. (2014). Characterization of the risks of adverse outcomes following rubella infection in pregnancy. Risk Anal 5, 1–17. 10.1111/risa.12264 25100307

[B106] Tondury G. Smith D. W. (1966). Fetal rubella pathology. J. Pediatr. 68, 867–879. 10.1016/S0022-3476(66)80204-4 5949097

[B107] Tyler C. V. Schramm S. C. Karafa M. Tang A. S. Jain A. K. (2011). Chronic disease risk in young adults with autism spectrum disorder: forewarned is forearmed. Am. J. Intellect. Dev. Disabil. 116, 371–380. 10.1352/1944-7558-116.5.371 21905805

[B108] Ushida M. Katow S. Furukawa S. (2003). Congenital rubella syndrome due to infection after maternal antibody conversion with vaccine. Jpn. J. Infect. Dis. 56, 68–69. 12824690

[B109] VanBang N. VanAnh N. T. Van V. T. Thai T. T. VanThuong N. Khandaker G. (2014). Surveillance of congenital rubella syndrome (CRS) in tertiary care hospitals in Hanoi, Vietnam during a rubella epidemic. Vaccine 32, 7065–7069. 10.1016/j.vaccine2014 25444828

[B110] Wang L. N. Wang Y. F. Horne C. C. Shiao L. C. (1990). Congenital rubella infection: escape of one monozygotic twin with two amnions, one chorion, and a single placenta. J. Formos. Med. Assoc. 89, 30–33. 1973707

[B111] Weber B. Enders G. Schlosser R. Wegerich B. Koenig R. Rabenau H. (1993). Congenital rubella syndrome after maternal reinfection. Infection 21, 118–121. 10.1007/BF01710747 8491520

[B112] Webster W. (1998). Teratogen update: congenital rubella. Teratology 58, 13–23. 10.1002/(SICI)1096-9926(199807)58:1<13:AID-TERA5>3.0.CO;2-2 9699240

[B113] Wegiel J. Kuchna I. Nowicki K. Imaki H. Wegiel J. Marchi E. (2010). The neuropathology of autism: defects of neurogenesis and neuronal migration, and dysplastic changes. Acta Neuropathol. 119, 755–770. 10.1007/s00401-010-0655 20198484 PMC2869041

[B114] Weir R. K. Forghany R. Smith S. E. Patterson P. H. McAllister A. K. Schumann C. M. (2015). Preliminary evidence of neuropathology in nonhuman primates prenatally exposed to maternal immune activation. Brain Behav. Immun 48, 139–146. 10.1016/j.bbi.2015.03.009 25816799 PMC5671487

[B115] Whitney E. R. Kemper T. L. Rosene D. L. Bauma M. L. Blatt G. J. (2009). Density of cerebellar basket and stellate cells in autism: evidence for a late developmental loss of Purkinje cells. J. Neurosci. Res. 87, 2245–2254. 10.1002/jnr.22056 19301429 PMC2760265

[B116] Williams E. L. Casanova M. F. (2011). Above genetics: lessons from cerebral development in autism. Transl. Neurosci. 2, 106–120. 10.2478/s13380-011-0016-3 22523638 PMC3331673

[B117] Wilson J. G. Gavan J. A. (1967). Congenital malformations in nonhuman primates: spontaneous and experimentally induced. Anat. Rec. 158, 99–109. 10.1002/ar.1091580111 4382271

[B118] Wolinsky J. S. Waxham M. N. Hess J. L. Townsend J. J. Baringer J. R. (1982). Immunochemical features of a case of progressive rubella encephalitis. Clin. Exp. Immunol. 48, 359–366. 7105489 PMC1536479

[B119] Xu N. Li X. Zhong Y. (2015). Inflammatory cytokines: potential biomarkers of immunologic dysfunction in autism spectrum disorders. Mediators Inflamm. 2015, 531518. 10.1155/2015/531518 25729218 PMC4333561

[B120] Yoshikura H. (2014). Impact of population size on incidence of rubella and measles in comparison with that of other infectious diseases. Jpn. J. Infect. Dis. 67, 447–457. 10.7883/yoken.67.447 25410560

[B121] Zilbovicius M. Saitovitch A. Popa T. Rechtman E. Diamandis L. Chabane N. (2013). Autism, social cognition and superior temporal sulcus. Open J. Psychiatr. 3, 46–55. 10.4236/ojpsych.2013.32A008

