# Supplementary figures and images for: Does Rubella Cause Autism: A 2015 Reappraisal?
Source: Front Hum Neurosci. 2016 Feb 1;10:25. doi: 10.3389/fnhum.2016.00025 (PMC4734211; doi:10.3389/fnhum.2016.00025)

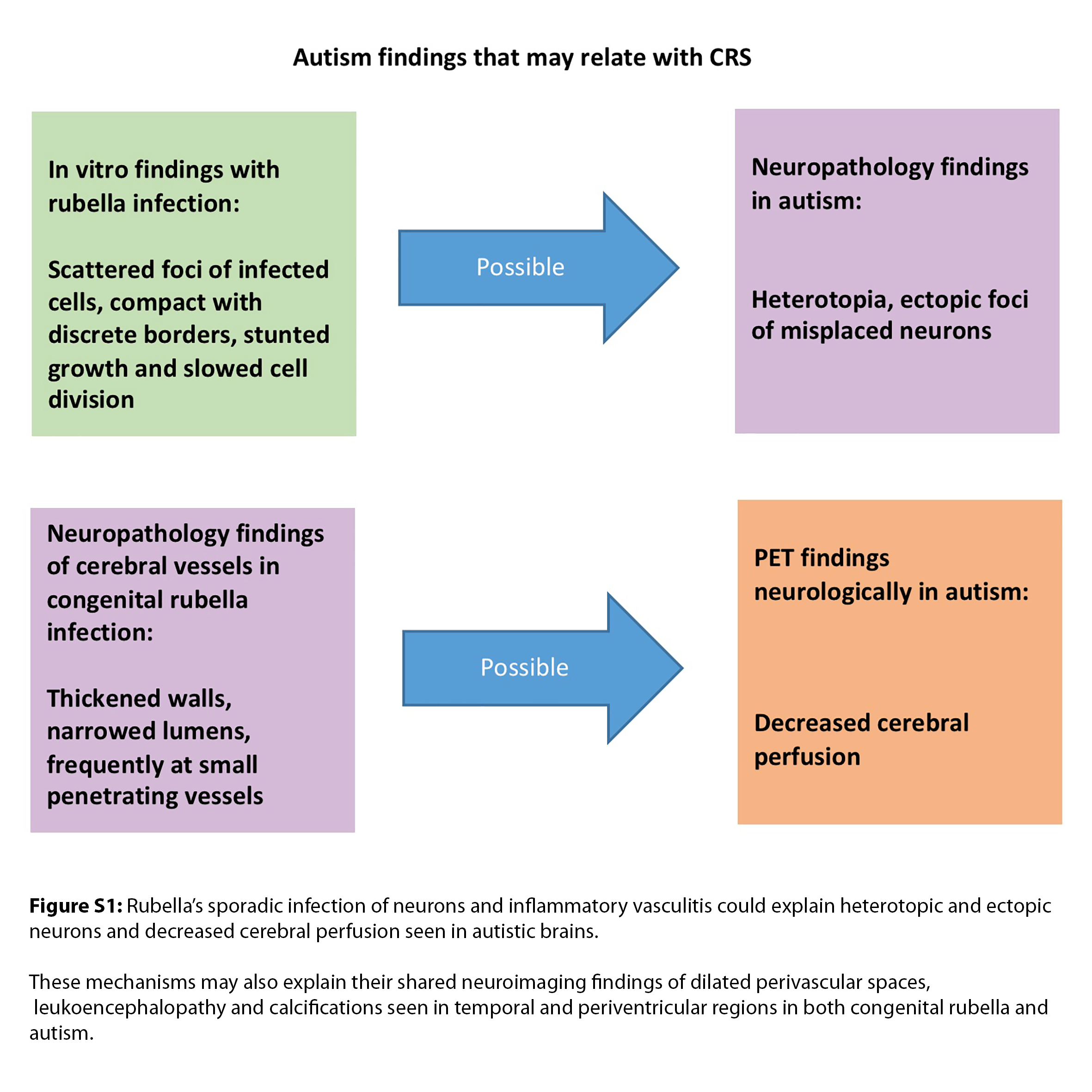

Supplement: Supplementary file 1 [file Image_1.TIF]
